# The increasing incidence and mortality of bacteremia in Denmark from 2010 to 2022: a population-based nationwide cohort study

**DOI:** 10.3389/fpubh.2024.1502893

**Published:** 2024-12-20

**Authors:** Ram B. Dessau, Christian Østergaard Andersen, John Coia, Svend Ellermann-Eriksen, Sophie Gubbels, Thøger Gorm Jensen, Jenny Dahl Knudsen, Jonas Kähler, Steen Lomborg, Lisbeth Lützen, Marc Trunjer Kusk Nielsen, Bente Scharvik Olesen, Mette Pinholt, Flemming Scheutz, Kirstine Kobberøe Søgaard, Marianne Voldstedlund, Kåre Mølbak

**Affiliations:** ^1^Department of Data Integration and Analysis, Statens Serum Institut, Copenhagen, Denmark; ^2^Department of Clinical Microbiology, Zealand University Hospital, Slagelse, Denmark; ^3^Department of Regional Health Research, University of Southern Denmark, Odense, Denmark; ^4^Department of Diagnostic and Infectious Disease Preparedness, Statens Seruminstitut, Copenhagen, Denmark; ^5^Department of Clinical Microbiology, Hvidovre University Hospital, Hvidovre, Denmark; ^6^Department of Regional Health Research, Research Unit of Clinical Microbiology, Esbjerg, Denmark; ^7^Department of Clinical Microbiology, Aarhus University Hospital, Aarhus, Denmark; ^8^Department of Clinical Microbiology, Odense University Hospital, Odense, Denmark; ^9^Department of Clinical Microbiology, Rigshospitalet, Copenhagen, Denmark; ^10^Department of Clinical Microbiology, Sygehus Sønderjylland, Aabenraa, Denmark; ^11^Department of Clinical Microbiology, Sygehus Lillebælt, Vejle, Denmark; ^12^Department of Clinical Microbiology, Herlev Hospital, Herlev, Denmark; ^13^The International Escherichia and Klebsiella Centre, Department of Bacteria, Parasites and Fungi, Statens Serum Institut, Copenhagen, Denmark; ^14^Department of Clinical Microbiology, Aalborg Hospital, Aalborg, Denmark; ^15^Department of Veterinary and Animal Science, Faculty of Health, University of Copenhagen, Copenhagen, Denmark; ^16^Epidemiological Infectious Disease Preparedness, Statens Serum Institut, Copenhagen, Denmark

**Keywords:** bacteremia, epidemiology, blood culture, aging adults, urinary tract infection

## Abstract

**Objectives:**

An increasing incidence of positive blood cultures has been reported in several countries. This represents an important challenge for public health due to the large number of hospital admissions with high mortality among these patients. This study aims to characterize the epidemiology and mortality associated with positive blood cultures.

**Study design and methods:**

We performed a nationwide cohort study, identifying all individuals with a blood culture drawn in Denmark from 2010 to 2022. We estimated the incidence of positive blood cultures and 30-day mortality per 100,000 individuals and modeled the case fatality rate (CFR) and population incidences with regression techniques.

**Results:**

We identified almost 4 million blood cultures drawn during the study period, increasing 64% from 220,757 in 2010 to 362,239 in 2022. During this period, the underlying population increased only 10%, whereas the population of older adults above 85 years increased 3.3 times. The positivity rate was 9.8%, and the most common pathogens were *Escherichia coli* (29%) and other pathogens associated with urinary tract infection, whereas *Staphylococcus aureus* accounted for 12%, and *Enterococcus faecium* for 4.1%. The 30-day CFR was 17% with a small reduction during the study period, with notable differences according to species.

**Conclusion:**

We found a substantial rise in blood culture activity and the incidence of positive cultures. This rise is driven by an increasing number of older citizens, which is expected to increase further in the coming years. Considering the high risk of mortality and increasing incidence, a national surveillance of bacteremia is warranted.

## Introduction

Detection of bacteria in blood samples is indicative of a severe infection with a high mortality. The incidence of positive blood cultures is more than 100 per 100,000 inhabitants per year, and an increasing incidence has been reported in Denmark and other countries (1–4). The majority of patients with bacteremia require hospital admission, and 30-day mortality is still as high as 12–17% (1, 5, 6). Comorbidity and advanced age are significant risk factors for bacteremia, and the increase in incidence is a major public health concern, particularly as projections indicate a substantial increase in the population aged over 65 years (7). In Denmark, continuous national-level surveillance is possible via the Danish Microbiology Database (MiBa) collecting results from all microbiological laboratories including blood cultures (8–12). The legislative framework is currently in place for the implementation of national surveillance of bacteremia. The present nationwide study aims to present key features of the epidemiology of blood culturing in Denmark using the DMiBa from 2010 to 2022. The study is intended to provide an informed basis for discussion and identify priorities for future work, particularly for international readers, as many countries are experiencing an aging population (13). The reader is referred to the companion article further elaborating on this topic (14).

## Methods

We performed a nationwide cohort study, covering all individuals with a blood culture drawn in Denmark in the period January 2010 to December 2022.

### Diagnostic settings and laboratory methods

All 5.9 million residents of Denmark have access to tax-financed public healthcare, with regional public departments of clinical microbiology serving all general physicians, outpatient clinics, and both public and private hospitals. Blood cultures are routinely performed during hospital admission on clinical suspicion of infection and sometimes in ambulatory care, for example in renal dialysis.

Standard methods for blood culture were used in all departments, utilizing either BACTEC^TM^ (Beckton Dickinson, Franklin Lakes, NJ, USA) or BACT/ALERT^TM^ (BioMérieux, France) systems. Pathogens are identified using conventional methods, including mass spectrometry and 16S rDNA PCR.

### Data sources and data

Statistics Denmark provided the gross population data to calculate incidences (15). The population count on 1 January of each year was used. The Danish Microbiology Database (MiBa) is a national database, that in real-time, receives copies of all microbiological test results from all Danish departments of clinical microbiology in a standardized format (8, 16, 17). Data on all blood cultures were extracted for the period 1 January 2010 to 31 December 2022, as defined by sampling date. Extracted variables included person identification, sample identification number, sampling date, and microorganisms identified. The Danish Civil Registrations System holds information such as civil registration number, sex, date of birth, date of death, migration status, place of residence, and family relations (7). Data on vital status were extracted and linked at the individual level to each episode by the national civil registration number until 30 January 2023.

### Definitions

The number of bottles included in a blood culture set differs among departments of clinical microbiology, and the number of blood culture sets taken per day per person differs over time and between clinical settings and circumstances (e.g., infectious endocarditis and follow-up blood culture). Therefore, a blood culture day was defined as a day on which a patient had at least one blood culture taken as defined previously (11).

Episodes of bacteremia were defined as first-time positive blood cultures with a specific species, and potentially included subsequent positive blood cultures with the same species within a 30-day period. If a different species was identified on the same day or later within the 30-day period, it was considered a new episode. Therefore, polybacteremia was counted as a separate episode. For this study, similar organisms were not grouped, and typical contaminants were not excluded. Thus, any reported “microorganism” was included, even those with general designations such as “Coagulase-negative staphylococci” and “Gram-negative rods.” This approach was taken as our study focused on exploring the overall occurrence of bacteremia episodes. The data extract also included episodes of fungemia; however, since none of these species appeared in the top 10 list, fungemia will not be further considered in this paper.

The 30-day all-cause mortality (case fatality rate, CFR) was estimated exclusively for the first episode for each individual, and if two episodes with different microorganisms were recorded on the same date, this was counted only once. For the 30-day mortality, we included the first month of 2023 to allow a 30-day follow-up for each episode, but only first-time episodes defined by sample date before 1 January 2023 were used in the statistical analysis. For the statistical modeling of the CFR and mortality, the first episode was chosen for 10 of the most frequent species. For the remaining individuals without any of the 10 most frequent species, any first event was chosen. For individuals with the detection of more than one species during the first episode, the species included in the analysis was randomly chosen by the software.

### Statistical methods

The R statistical package was used for statistical analysis and graphics (18). We estimated the annual incidence of positive blood cultures per 100,000 individuals and calculated 30-day mortality. A logistic regression model was constructed to assess variables influencing the 30-day CFR, and a Poisson regression with robust confidence intervals was used for the incidence with the total population as offset variable. For the modeling, age was grouped into the following categories: 0–64, 65–74, 75–84, and 85 years and above. This age grouping focusing on the population above 65 years of age is similar to a recent publication from the Danish Ministry of Health showing the demographic development to be used for government policy (19). Fewer groups of bacterial species were also used.

## Results

### Blood cultures

**Figure 1:**
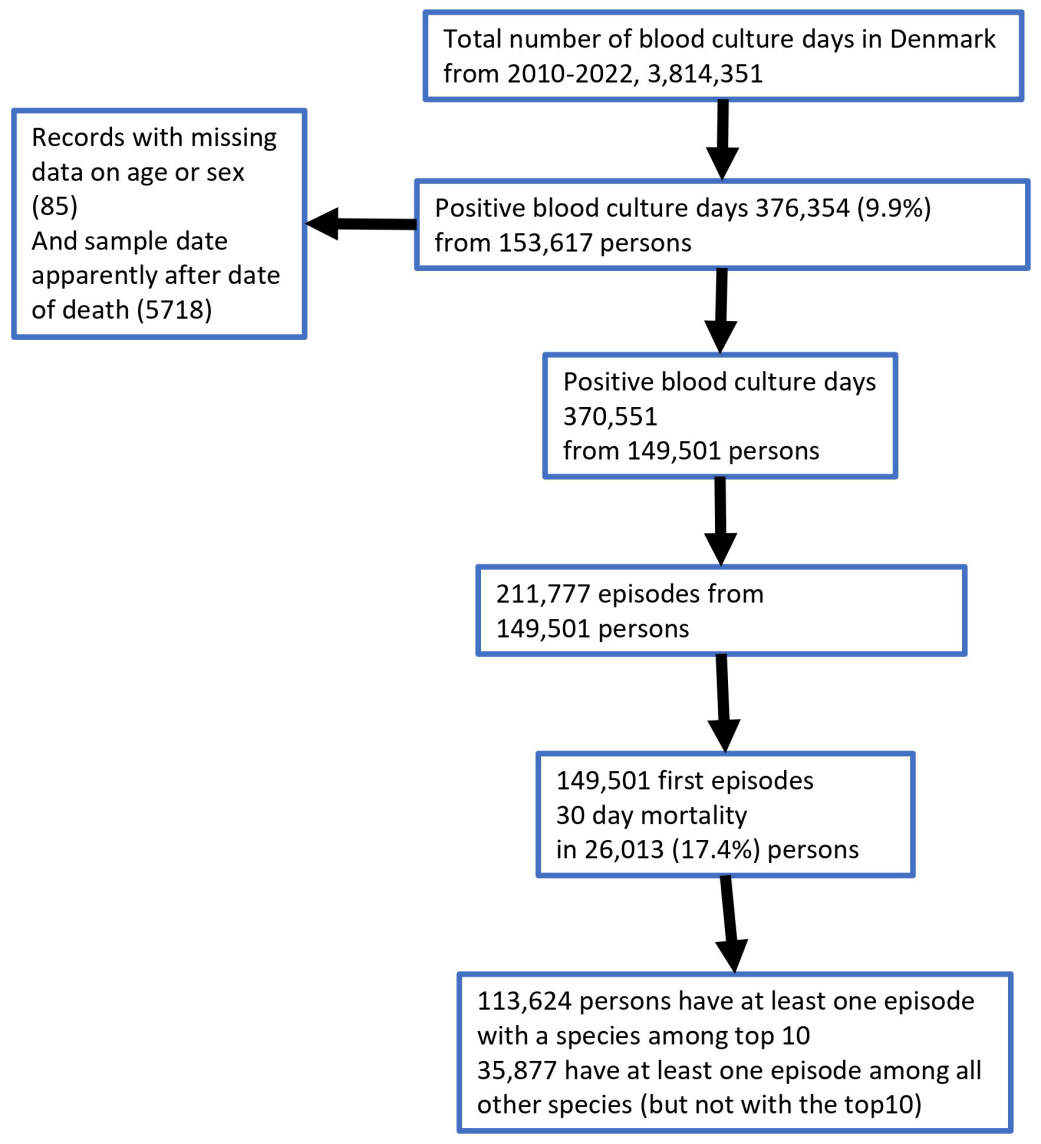
Flow diagram of the dataset.

**Figure 2:**
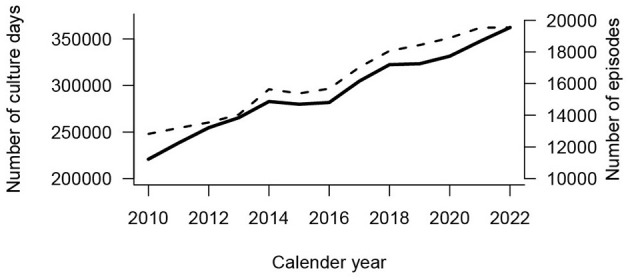
Number of blood culture days (*n* = 3,814,351) for each year (solid line, left *y*-axis) and number of episodes with growth (*n* = 211,777, broken line, right *y*-axis).

The total number of blood culture days from 2010 to 2022 was ~4 million (Figure 1) of which 376,356 (9.8%) showed growth. The number of blood culture days increased from 220,757 in 2010 to 362,239 in 2022 (Figure 2). The number of culture-positive episodes was 211,777 from 149,501 unique individuals, with 64,964 (43.5%) women and 84,537 (56.5%) men. The number of episodes followed a similar rising trend as the number of blood culture days (Figure 2) increasing from 12,820 in 2010 to 19,550 in 2022. Among the 211,777 episodes, the number for each individual ranged from 1 to 52 episodes, with 75% of individuals having only one episode, 16% having two episodes, 5% having 3 episodes, 2% having four episodes, and 2% having five or more episodes. The dataset of 211,777 episodes was used for the subsequent data analysis (Figure 1).

### Age and incidence

**Figure 3:**
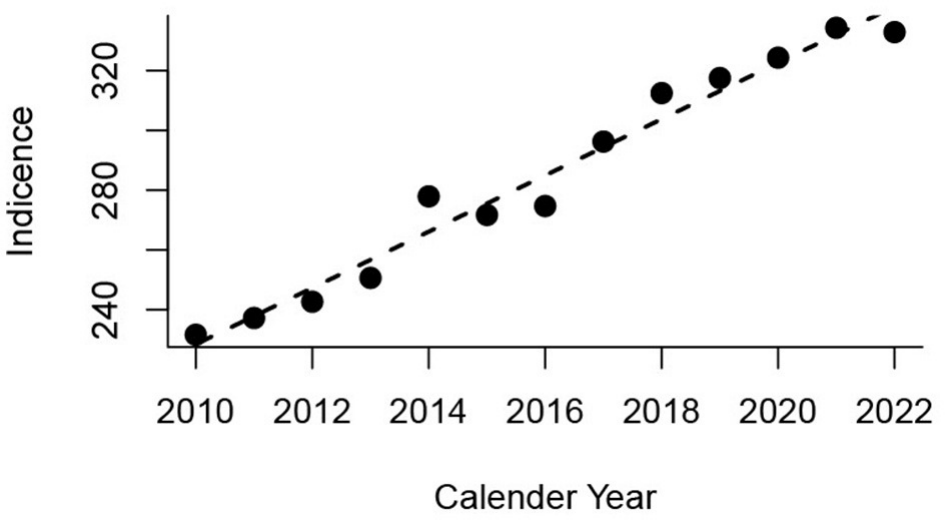
Incidence of episodes with bacteremia per 100,000 total population per year. The linear model (broken line) indicates an average rise of 9.4 episodes per l00,000 per year (95% CI 8.3–10.5).

**Figure 4:**
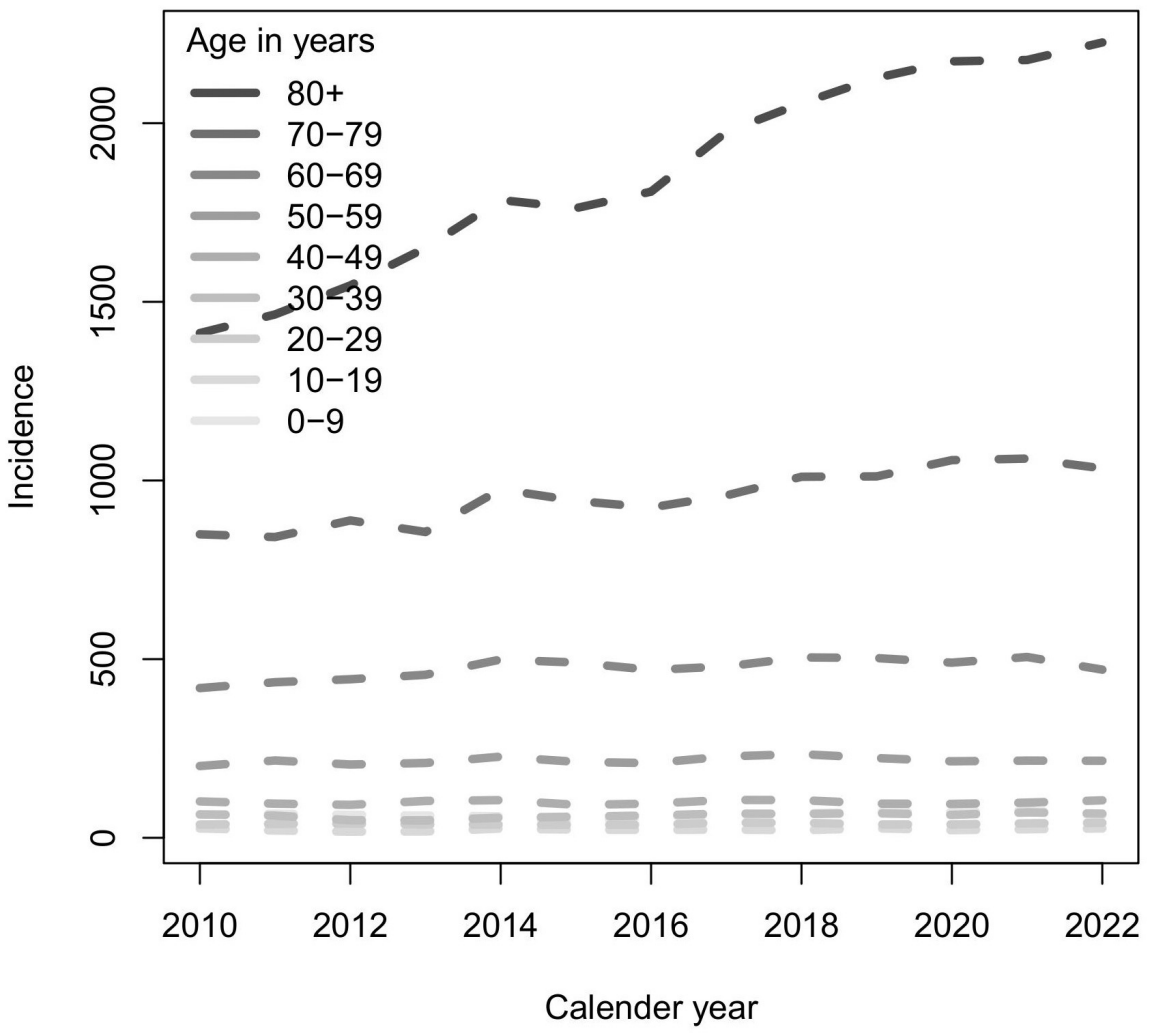
Age groups and incidence of episodes of bacteremia per 100,000 population for each year from 2010 to 2022.

**Figure 5:**
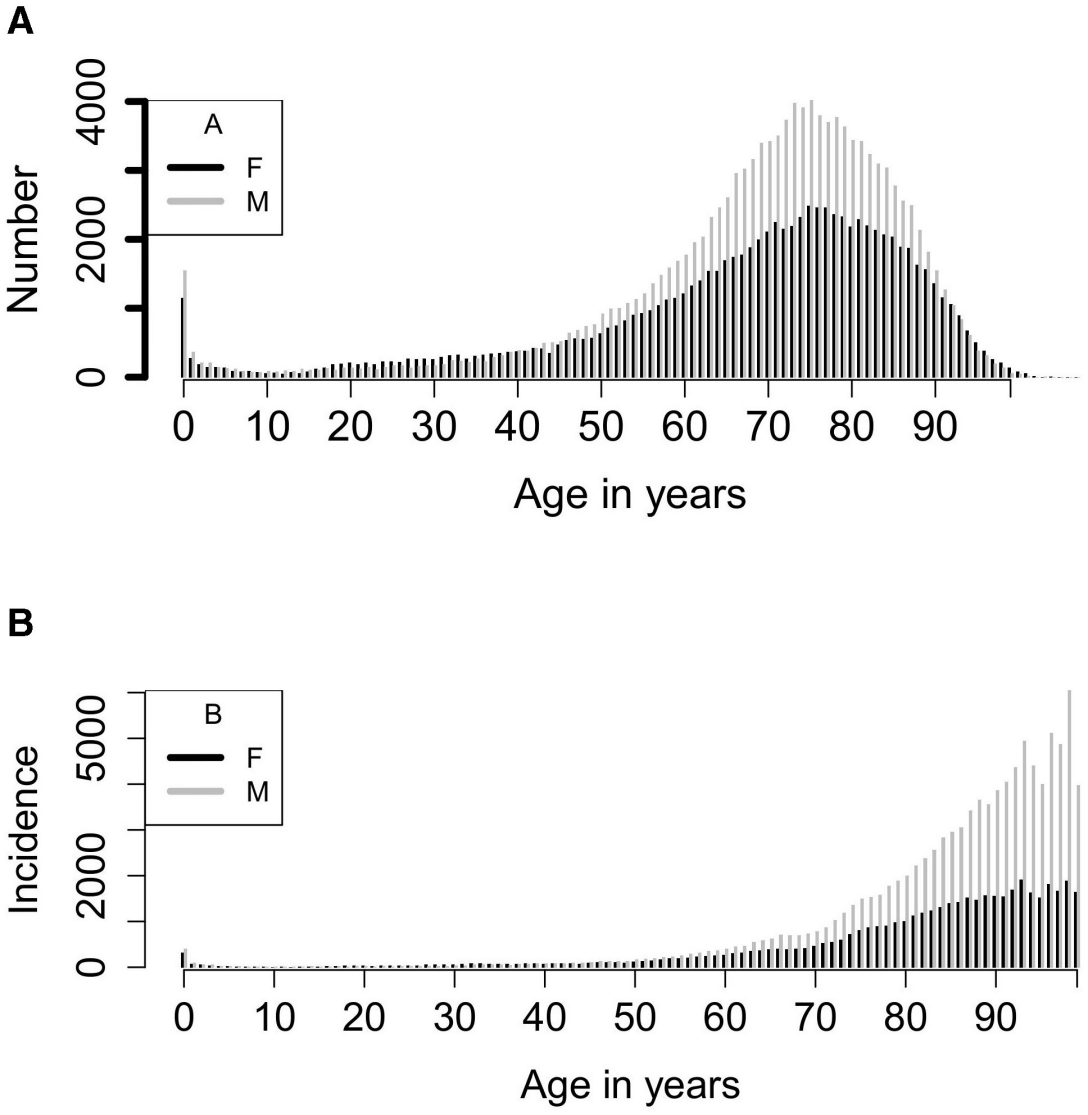
**(A)** Number of episodes for each year of age from 0 to 109 years. **(B)** Incidence of episodes for each year of age per 100,000 population. Calculated as 100,000* (total episodes for each year of age/13)/population of the first quarter of the year 2016. Numbers for ages 99 years and above have been added together.

The total Danish population increased from 5.2 to 5.9 million from 2010 to 2022 (1.1 times). However, from 2010 to 2022, there was a rise in the Danish population aged 65–84 years from 792,896 to 1,064567 (1.3 times) and 85 years and above from 109,963 to 366,207 (3.3 times). The incidence of episodes per 100,000 population increased from 232 to 333 per 100,000, reflecting an average linear increase in incidence of 9.4 per year (Figure 3). This rise was partly driven by population dynamics. While there was little change in incidence among younger age groups, a clear increase was observed in individuals aged 80 years and older (Figure 4), with the total number of episodes doubling from 3,214 episodes in 2010 to 6,488 in 2022. The number of episodes peaked at 75 years of age with 4,021 episodes for men and 2,488 for women (Figure 5A). The age-specific incidence per 100,000 population showed a marked increase and reached a peak of ~5,000 for the oldest male age groups, while for women, the peak was < 2,000 per 100,000 population (Figure 5B).

### Distribution of organisms

**Figure 6:**
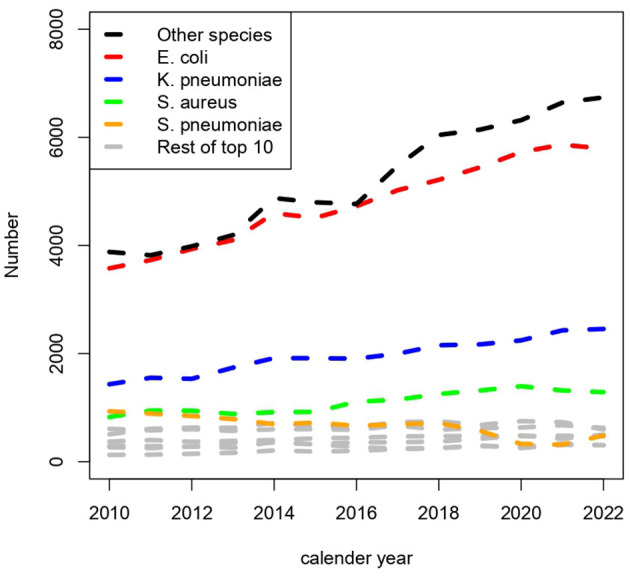
Number of episodes of bacteremia per year and the distribution of species.

A total of 750 different organisms were detected. The 10 most frequent microorganisms accounted for 144,100 (68%) of the episodes with *Escherichia coli* as the far most frequent with 29% of all episodes (Table 1). Time trends for the 10 most frequent organisms showed that *E. coli, Klebsiella pneumoniae*, and *Staphylococcus aureus* were the main contributors to the rising number of episodes (Figure 6). *Enterococcus faecium* was also a common finding. Species often causing urinary tract infections constituted 52% of all species (Table 1). Of note, episodes with *Streptococcus pneumonia* declined from 932 in 2010 to 483 in 2022.

**Table 1:** Ten most frequent microorganisms detected in 144,100 of the 211,777 episodes of bacteremia in Denmark from 2010 to 2022.

**Microorganism**	**Number of episodes (%)**
*Escherichia coli*	62,237 (29.4)^*^
*Staphylococcus aureus*	25,442 (12.0)
*Klebsiella pneumoniae*	14,254 (6.7)^*^
*Streptococcus pneumoniae*	8,633 (4.1)
*Enterococcus faecium*	8,624 (4.1)^*^
*Enterococcus faecalis*	7,958 (3.8)^*^
*Pseudomonas aeruginosa*	5,633 (2.7)^*^
*Klebsiella oxytoca*	4,740 (2.2)^*^
*Enterobacter cloacae*	3,772 (1.8)^*^
*Proteus mirabilis*	2,807 (1.3)^*^
Total	144,100 (68.0%)

^*^Species often associated with urinary tract infection: 52%.

### Mortality

**Figure 7:**
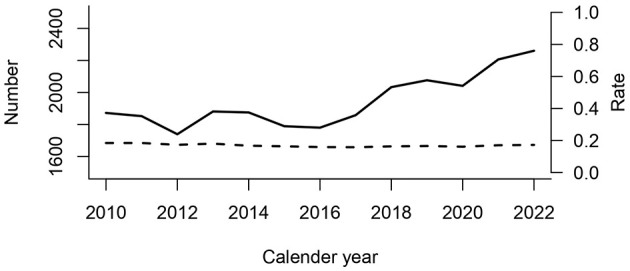
Number of individuals with 30-day mortality after the first recorded episode per year (solid line). The 30-day case fatality rate per first recorded episode for each year is also shown (broken line/right *y*-axis).

Among 149,501 individuals eligible for the mortality analysis, 26,013 died within 30 days, corresponding to a CFR of 17.4% (Figure 1). Figure 7 shows that the annual number of deaths increased from 1,872 in 2016 to 2,260 in 2022.

In a multivariable logistic regression model, we found no difference in 30-day CFR after the first episode between men and women (Table 3). To illustrate differences according to species, we used *E. coli* as a reference for the other bacteria. We found that other *Enterobacterales, P. aeruginosa*, and *S. aureus* were associated with higher OR ranging from 1.35 to 2.26. The highest relative mortality (OR = 4.68) was found in *E. faecium*. In contrast, *S. pneumoniae* had a similar CFR (OR 1.16) as *E. coli*. The CFR from 2010 to 2022 (OR 0.98, *p* < 0.001) indicated an average decline of 2% per year when adjusted for age and sex.

To analyze mortality in relation to the total population a Poisson regression model of 30-day mortality in 26,013 individuals after a first episode was constructed (Table 2; Figure 1). The bacterial species were grouped into fewer groups, to avoid subgroups with small tabulated counts according to the four variables sex, age group, cultured species, and year of episodes. The mortality rate ratio (MRR) was highest among the oldest age groups and lower among less frequent species, in particular *S. pneumoniae*. The model shows that there was no significant time trend in the mortality rate despite the increasing number of episodes. This indicates that the demographic development, with more senior citizens, contributed to the rise in the number of episode-related deaths. Men had a higher incidence (MRR of 1.78) than women.

**Table 2:** Thirty-day mortality rate ratio (MRR) in the population in a Poisson regression model with data tabulated on the year of first episode, sex, and age groups for 26,013 patients and with the underlying population as an offset variable.

**Species**	**MRR**	**95% CI**
*Escherichia coli*	Reference	
All other species	1.02	0.97, 1.08
Rest of the top 10	0.79	0.15, 0.84
*Klebsiella pneumoniae*	0.26	0.24, 0.28
*Staphylococcus aureus*	0.72	0.68, 0.77
*Streptococcus pneumoniae*	0.16	0.14, 0.18
**Age group**		
65–74	Reference	
0–64	0.10	0.09, 0.10
65–84	2.54	2.40, 2.68
85 and above	6.51	6.16, 6.88
**Sex**		
Female	Reference	
Male	1.78	1.71, 1.85
**Year from 2010 to 2022**		
Year of first episode	1.00	0.99, 1.00

MRR, mortality rate ratio; CI, confidence interval.

The bacterial species and age intervals are grouped into fewer groups to avoid small counts.

## Discussion

Denmark has extensive data on healthcare events, which makes research based on the entire population possible (8, 9). Our study found a linear rise from 249 to 360 blood culture episodes per 100,000 population from 2010 to 2023. Individuals older than 70 years were the main contributors, and *E. coli* was the most frequently isolated species, contributing substantially to the increase in episodes. The CFR was ~17%, and this rate remained stable during the study period. The increasing incidence of mortality is mainly driven by a larger number of older individuals in the population above 75 years. We observed that older men had a notably large burden of bacteremia episodes. The fact that this part of the population is expected to increase in numbers over the coming years, calls for increased attention toward prevention of underlying infections such as urinary tract infections. A study from 1992 to 2006 also documented an increase in the incidence of positive blood culture episodes from 114 to 166 per 100,000 inhabitants (20). Thus, this unfortunate trend has been seen for at least 30 years. The rising trend in the older population was also observed in a previous study using MiBa over 4 years from 2010 to 2013 (11). In contrast, a study from Funen County in Denmark documented a decrease in the overall incidence rate during 2000–2008 from 254 to 199 (21). In the present study a decline in incidence of 2% per year was found that may be translated into a 25% decline over the study period. The reason for the lower incidence compared to the 249 per 100,000 found in our 2010 data remains unclear. However, the establishment and organization of emergency departments since 2007 may have been considerably strengthened, and this could contribute to an increase in the number of blood cultures (22, 23). Emergency departments have standardized routine procedures, including blood cultures, based on sepsis guidelines, which have contributed to an increased use of blood cultures. Similar trends and incidence levels of bacteremia reaching above 200 per 100,000 of incidence of bacteremia have been found in neighboring countries (1, 3, 6). Concerning case fatality, a previous study from Denmark using data from two regions observed a decrease in the CFR from 22.7% in 2000 to 17.4% in 2014 (23). The crude CFR (Figure 7) indicates that case fatality has stabilized at ~17%. However, old age is a major determinant for mortality, and the increasing number of older individuals with bacteremia must be considered in the interpretation of the case fatality. Thus, the multivariable model presented in Table 3 shows that case fatality declined when age and other confounders were included. The decline of 2% per year may be translated into a 25% decline over the study period. The burden of nosocomial episodes has also been a focus surveillance, and in Denmark the HAIBA system was developed. The HAIBA system uses a register-based algorithm counting hospital-acquired blood culture events after hospital admission (24). The burden of hospital-acquired bacteremia was relatively smaller than the total number of episodes. In 2022, there were 1,277 events detected by HAIBA corresponding to an incidence of 23 per 100,000 population in Denmark (25). Thus, the burden of episodes is increasing in Denmark and other countries. This calls for routine surveillance and research to identify causes and targets for intervention.

**Table 3:** Odds ratios (ORs) from the logistic regression model of 30-day case fatality (CFR) after the first episode (n = 149,501) among the 10 most frequent organisms or any first episode among individuals not having an episode caused by the 10 most frequently identified organisms.

**Characteristic**	**OR**	**95% CI**
**Sex**		
Female	Reference	
Male	1.00	0.97, 1.03
**Age group**		
65–74	Reference	
0–64	0.53	0.51, 0.56
65–84	1.34	1.30, 1.39
85 and above	2.21	2.12, 2.30
**Species**		
*Escherichia coli*	Reference	
All other species	1.78^a^	1.71, 1.85
*Enterobacter cloacae*	1.64	1.48, 1.82
*Enterococcus faecalis*	1.32	1.22, 1.42
*Enterococcus faecium*	4.68	4.40, 4.98
*Klebsiella oxytoca*	1.55	1.41, 1.70
*Klebsiella pneumoniae*	1.43	1.35, 1.52
*Proteus mirabilis*	1.35	1.20, 1.52
*Pseudomonas aeruginosa*	1.89	1.74, 2.05
*Staphylococcus aureus*	2.26	2.17, 2.36
*Streptococcus pneumoniae*	1.16	1.08, 1.24
Years from 2010 to 2022	0.98^b^	0.98, 0.98

OR, odds ratio; CI, confidence interval.

^a^This OR is selected of any first event in individuals without an episode among the top 10 species (see Methods Section).

^b^Corresponding to a 25% reduction in CFR from 2010 to 2022.

In these raw numbers from the MiBa database, potential contaminants, such as skin flora-type coagulase-negative staphylococci, were not excluded from the episodes. As a combined group, these would be quite frequent, but as individual species, the numbers were low and did not reach the top 10 most frequent species. The present study counts polybacteremia as separate episodes and includes potential contaminants in contrast to many other studies. Contaminants have been found to constitute 17.7% of cases in Denmark (26). Excluding potential contaminants also risks excluding episodes from patients with hemodialysis, implanted devices, and parental feeding. The main purpose of this study was to highlight the overall rising trend in blood culture events and not to explore details. The study points to aspects needing further analysis and surveillance in subsequent studies exploring these details. The episodes with *E. coli* and other enterobacteriaceae are very frequent, especially in older men, and urinary tract infections could be proposed as a target for prevention. Firm conclusions on these issues need further exploration. All-cause 30-day mortality is used ubiquitously because it is a practical and easy statistic and allows for comparability between studies. However, setting such an administrative cutoff of 30 days may be problematic, and results may vary by manipulating such a cut-point (27–29).

## Conclusion

We found an increase in the number of blood cultures taken and in bacteremia episodes from 2010 to 2022. This increase was substantial in the older population above 70 years. *E. coli* and other Enterobacteriaceae associated with urinary tract infections constitute 52% of all episodes. The all-cause 30-day CFR after the first registered episode was 17%. The development in the epidemiology of blood culture episodes mainly follows the increasingly older population in Denmark with a higher incidence in the male population. Our study underpins that bacteremia represents a significant burden for public health. The availability of national electronic health records will facilitate the development of surveillance for this condition.

## Data Availability

The datasets presented in this article are not readily available because the original data is part of surveillance obligations of the Statens Serum Institute in Denmark. It is at the outset not anonymized, nor freely available. Application for research access is possible. Requests to access the datasets should be directed to https://miba.ssi.dk/forskningsbetjening.
